# Marbofloxacin

**DOI:** 10.1107/S1600536812009312

**Published:** 2012-03-10

**Authors:** Jin Shen, Jing-Jing Qian, Jian-Ming Gu, Xiu-Rong Hu

**Affiliations:** a College of Pharmaceutical Science, Zhejiang Chinese Medical University, Hangzhou, Zhejiang 310053, People’s Republic of China; b Center of Analysis and Measurement, Zhejiang University, Hangzhou, Zhejiang 310028, People’s Republic of China

## Abstract

In the title compound, [systematic name: 9-fluoro-2,3-dihydro-3-methyl-10-(4-methyl­piperazin-1-yl)-7-oxo-7*H*-pyrido[1,2,3-*ij*][1,2,4]benzoxadiazine-6-carb­oxy­lic acid], C_17_H_19_FN_4_O_4_, the carbonyl and carboxyl groups are coplanar with the quinoline ring, making a dihedral angle of 2.39 (2)°. The piperazine ring adopts a chair conformation and the oxadiazinane ring displays an envelope conformation with the CH_2_ group at the flap displaced by 0.650 (2) Å from the plane through the other five atoms. The mol­ecular structure exhibits an *S*(6) ring motif, owing to an intra­molecular O—H⋯O hydrogen bond. In the crystal, weak C—H⋯F hydrogen bonds link mol­ecules into layers parallel to the *ab* plane.

## Related literature

Marbofloxacin is a third-generation fluoro­quinolone for veterinary use, the anti­microbial activity of which depends upon its inhibition of DNA-gyrase and topoisomerase IV (Paradis *et al.*, 2001 ▶; Thomas *et al.*, 2001 ▶; Voermans *et al.*, 2006 ▶). With a broad spectrum bactericidal activity and good efficacy, marbofloxacin is indicated for dermatological, respiratory and urinary tract infections resulting from both Gram-positive and Gram-negative bacteria (Lefebvre *et al.*, 1998 ▶) and Mycoplasma (Spreng *et al.*, 1995 ▶; Dossin *et al.*, 1998 ▶; Carlotti *et al.*, 1999 ▶; Ishak *et al.*, 2008 ▶).
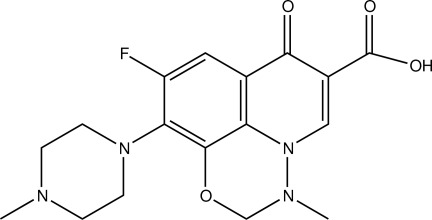


## Experimental

### 

#### Crystal data

C_17_H_19_FN_4_O_4_
*M* *_r_* = 362.36
Triclinic, 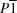

*a* = 8.0145 (5) Å
*b* = 8.9218 (6) Å
*c* = 13.0874 (8) Å
α = 91.65 (3)°
β = 99.65 (3)°
γ = 115.091 (10)°
*V* = 830.26 (16) Å^3^
*Z* = 2
Mo *K*α radiation
μ = 0.11 mm^−1^
*T* = 296 K
0.31 × 0.13 × 0.03 mm

#### Data collection

Rigaku RAXIS-RAPID/ZJUG diffractometer
Absorption correction: multi-scan (Higashi, 1995 ▶) *T*_min_ = 0.956, *T*_max_ = 0.997
6601 measured reflections
2925 independent reflections
1428 reflections with *I* > 2σ(*I*)
*R*_int_ = 0.052

#### Refinement

*R*[*F* ^2^ > 2σ(*F* ^2^)] = 0.055
*wR*(*F* ^2^) = 0.203
*S* = 1.00
2925 reflections
241 parameters
1 restraint
H atoms treated by a mixture of independent and constrained refinement
Δρ_max_ = 0.29 e Å^−3^
Δρ_min_ = −0.34 e Å^−3^

### 

Data collection: *PROCESS-AUTO* (Rigaku, 2006 ▶); cell refinement: *PROCESS-AUTO*; data reduction: *CrystalStructure* (Rigaku, 2007 ▶); program(s) used to solve structure: *SHELXS97* (Sheldrick, 2008 ▶); program(s) used to refine structure: *SHELXL97* (Sheldrick, 2008 ▶); molecular graphics: *ORTEP-3 for Windows* (Farrugia, 1997 ▶); software used to prepare material for publication: *WinGX* (Farrugia, 1999 ▶).

## Supplementary Material

Crystal structure: contains datablock(s) global, I. DOI: 10.1107/S1600536812009312/nr2019sup1.cif

Structure factors: contains datablock(s) I. DOI: 10.1107/S1600536812009312/nr2019Isup2.hkl

Supplementary material file. DOI: 10.1107/S1600536812009312/nr2019Isup3.cml

Additional supplementary materials:  crystallographic information; 3D view; checkCIF report

## Figures and Tables

**Table 1:** Hydrogen-bond geometry (Å, °)

*D*—H⋯*A*	*D*—H	H⋯*A*	*D*⋯*A*	*D*—H⋯*A*
O1—H1⋯O3	0.83 (3)	1.77 (2)	2.560 (4)	159 (5)
C12—H12*B*⋯F1^i^	0.96	2.62	3.422 (3)	140 (4)
C15—H15*B*⋯F1^ii^	0.97	2.54	3.446 (3)	155 (5)

Symmetry codes: (i) 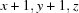; (ii) 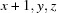.

**Fig. 1.**
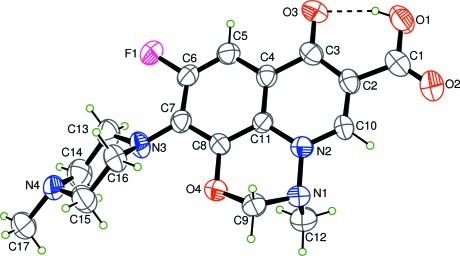
Molecular structure of marbofloxacin showing atom-labelling scheme and displacement ellipsoids at 40% probability level. H atoms are shown as small circles of arbitary radii.

**Fig. 2.**
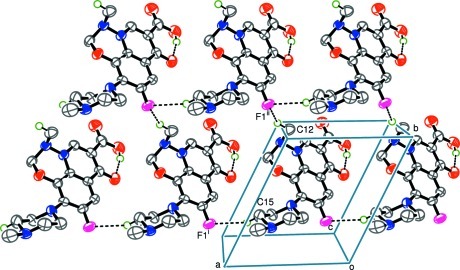
Part of the crystal packing of Marbofloxacin. Weak Hydrogen bonds are shown as dashed lines. H atoms not involved in hydrogen bonding have been omitted for clarity. Symmetric code: (i)1 + x,y,z; (ii)1 + x,1 + y,z.

## supplementary crystallographic information

### Comment

Marbofloxacin is a third-generation fluoroquinolone for veterinary use, the antimicrobial of which depends upon its inhibition of DNA-gyrase and topoisomerase IV (Paradis *et al.*, 2001; Thomas *et al.*, 2001; Voermans *et al.*, 2006). With a broad spectrum bactericidal activity and good efficacy, marbofloxacin is indicated for dermatological, respiratory and urinary tract infections due to both Gram-positive and Gram-negative bacteria (Lefebvre *et al.*, 1998) and Mycoplasma (Spreng *et al.*, 1995; Dossin *et al.*, 1998; Carlotti *et al.*, 1999; Ishak *et al.*, 2008). But up till now, no single-crystal structure of marbofloxacin has been reported. In the prestent study, we report the crystal structure of marbofloxacin, recrystallized from methanol.

In the crystal structure of marbofloxacin (Fig.1), the carbonyl and carboxyl group are coplanar with the quinoline ring system. The least-squares plane through atoms O1, C1, C2, C3 and O3 is rotated by 2.39 (2)° with respect to the least-scqures plane of quinolinemoiety. The quionline moiety is planar, with the maximum displacement from the least-squares plane being observed for atom C2 [0.020 Å].

The piperazine ring adopts a chair conformation, with the distance of 0.663 (7) Å, -0.662 (7) Å for N4 and N3 to the plane of C13, C14, C15, C16, respectively. The oxadiazinane ring diaplays an envelop conformation. The methyl substituent on N1 is perpendicular to the quinoline moiety, with a C12—N1—N2—C10 torsion angle of -88.8 (4)°.

The carboxyl atom O1 and carbonyl atom O3 is connected by intramolecular hydrogen bond O1—H1···O3 and formed a six-membered ring. Weak intermolecular C15—H15B···F1^i^[Symmetric code:(i)1 + *x*,*y*,*z*] interaction link molecules into chains along *a* axis, which is stacked along *b* axis through another weak intermolecular interaction C12—H12B···F1^ii^[Symmetric code:(ii)1 + *x*,1 + *y*,*z*].

### Experimental

The crude product is supplied by Zhejiang Excel Pharmaceutical Co.,Ltd. It was recrystallized from methanol solution, giving yellow crystal of marbofloxacin suitable for X-ray diffraction.

### Refinement

Atom H1 was placed from the difference fourier density and refined free with restraints to the OH bond of O1—H1=0.82 (1) Å. All other H atoms were placed in calculated positions with C—H = 0.93–0.97 Å and included in the refinement in riding model, with *U*_iso_(H) = 1.2*U*_eq_ or 1.5*U*_eq_(carrier atom).

### Crystal data

**Table:**

C_17_H_19_FN_4_O_4_	*Z* = 2
*M**_r_* = 362.36	*F*(000) = 380
Triclinic, *P*1	*D*_x_ = 1.449 Mg m^−^^3^
Hall symbol: -P 1	Mo *K*α radiation, λ = 0.71073 Å
*a* = 8.0145 (5) Å	Cell parameters from 4017 reflections
*b* = 8.9218 (6) Å	θ = 3.1–27.4°
*c* = 13.0874 (8) Å	µ = 0.11 mm^−^^1^
α = 91.65 (3)°	*T* = 296 K
β = 99.65 (3)°	Plates, yellow
γ = 115.091 (10)°	0.31 × 0.13 × 0.03 mm
*V* = 830.26 (16) Å^3^	

### Data collection

**Table:**

Rigaku RAXIS-RAPID/ZJUG diffractometer	2925 independent reflections
Radiation source: rolling anode	1428 reflections with *I* > 2σ(*I*)
Graphite monochromator	*R*_int_ = 0.052
Detector resolution: 10.00 pixels mm^-1^	θ_max_ = 25.0°, θ_min_ = 3.1°
ω scans	*h* = −9→9
Absorption correction: multi-scan (Higashi, 1995)	*k* = −10→10
*T*_min_ = 0.956, *T*_max_ = 0.997	*l* = −15→15
6601 measured reflections	

### Refinement

**Table:**

Refinement on *F*^2^	Primary atom site location: structure-invariant direct methods
Least-squares matrix: full	Secondary atom site location: difference Fourier map
*R*[*F*^2^ > 2σ(*F*^2^)] = 0.055	Hydrogen site location: inferred from neighbouring sites
*wR*(*F*^2^) = 0.203	H atoms treated by a mixture of independent and constrained refinement
*S* = 1.00	*w* = 1/[σ^2^(*F*_o_^2^) + (0.072*P*)^2^ + 0.8525*P*] where *P* = (*F*_o_^2^ + 2*F*_c_^2^)/3
2925 reflections	(Δ/σ)_max_ < 0.001
241 parameters	Δρ_max_ = 0.29 e Å^−^^3^
1 restraint	Δρ_min_ = −0.34 e Å^−^^3^

### Special details

**Table:**

Geometry. All e.s.d.'s (except the e.s.d. in the dihedral angle between two l.s. planes) are estimated using the full covariance matrix. The cell e.s.d.'s are taken into account individually in the estimation of e.s.d.'s in distances, angles and torsion angles; correlations between e.s.d.'s in cell parameters are only used when they are defined by crystal symmetry. An approximate (isotropic) treatment of cell e.s.d.'s is used for estimating e.s.d.'s involving l.s. planes.
Refinement. Refinement of *F*^2^ against ALL reflections. The weighted *R*-factor *wR* and goodness of fit *S* are based on *F*^2^, conventional *R*-factors *R* are based on *F*, with *F* set to zero for negative *F*^2^. The threshold expression of *F*^2^ > σ(*F*^2^) is used only for calculating *R*-factors(gt) *etc*. and is not relevant to the choice of reflections for refinement. *R*-factors based on *F*^2^ are statistically about twice as large as those based on *F*, and *R*- factors based on ALL data will be even larger.

### Fractional atomic coordinates and isotropic or equivalent isotropic displacement parameters (Å^2^)

**Table:**

	*x*	*y*	*z*	*U*_iso_*/*U*_eq_	
F1	0.2105 (3)	0.1945 (3)	0.64667 (19)	0.0642 (7)	
O1	0.2871 (5)	0.7762 (4)	1.0967 (2)	0.0680 (9)	
H1	0.230 (6)	0.693 (4)	1.053 (3)	0.09 (2)*	
O2	0.5831 (5)	0.9686 (4)	1.1352 (2)	0.0732 (10)	
O3	0.1921 (4)	0.5352 (4)	0.9550 (2)	0.0594 (8)	
O4	0.8358 (3)	0.6152 (3)	0.7475 (2)	0.0539 (8)	
N1	0.9081 (4)	0.8423 (4)	0.8761 (3)	0.0499 (9)	
N2	0.7233 (4)	0.7572 (4)	0.8983 (2)	0.0455 (8)	
N3	0.5854 (4)	0.3404 (4)	0.6136 (2)	0.0494 (9)	
N4	0.7031 (5)	0.1924 (4)	0.4634 (3)	0.0551 (9)	
C1	0.4644 (7)	0.8439 (6)	1.0818 (3)	0.0576 (11)	
C2	0.5010 (5)	0.7549 (5)	0.9964 (3)	0.0451 (10)	
C3	0.3568 (6)	0.6070 (5)	0.9367 (3)	0.0467 (10)	
C4	0.4097 (5)	0.5369 (4)	0.8514 (3)	0.0414 (9)	
C5	0.2802 (5)	0.3970 (5)	0.7853 (3)	0.0475 (10)	
H5	0.1554	0.3464	0.7926	0.057*	
C6	0.3386 (5)	0.3344 (5)	0.7091 (3)	0.0484 (10)	
C7	0.5238 (5)	0.4014 (4)	0.6918 (3)	0.0436 (9)	
C8	0.6516 (5)	0.5420 (5)	0.7594 (3)	0.0441 (9)	
C9	0.9600 (5)	0.7160 (5)	0.8421 (3)	0.0557 (11)	
H9A	0.9574	0.6446	0.8969	0.067*	
H9B	1.0876	0.7688	0.8302	0.067*	
C10	0.6778 (5)	0.8265 (5)	0.9749 (3)	0.0463 (10)	
H10	0.7678	0.9247	1.0140	0.056*	
C11	0.5951 (5)	0.6118 (4)	0.8364 (3)	0.0400 (9)	
C12	0.9089 (7)	0.9603 (5)	0.7983 (4)	0.0665 (13)	
H12A	0.8252	0.8996	0.7343	0.100*	
H12B	1.0339	1.0196	0.7856	0.100*	
H12C	0.8681	1.0380	0.8246	0.100*	
C13	0.4605 (6)	0.2587 (6)	0.5132 (3)	0.0628 (12)	
H13A	0.3799	0.3129	0.4920	0.075*	
H13B	0.3817	0.1428	0.5190	0.075*	
C14	0.5809 (7)	0.2709 (6)	0.4340 (3)	0.0684 (13)	
H14A	0.5007	0.2181	0.3667	0.082*	
H14B	0.6560	0.3872	0.4272	0.082*	
C15	0.8245 (6)	0.2699 (6)	0.5640 (3)	0.0651 (13)	
H15A	0.9056	0.3855	0.5587	0.078*	
H15B	0.9034	0.2138	0.5841	0.078*	
C16	0.7106 (6)	0.2613 (5)	0.6461 (3)	0.0546 (11)	
H16A	0.6367	0.1459	0.6557	0.065*	
H16B	0.7939	0.3180	0.7120	0.065*	
C17	0.8116 (7)	0.1965 (6)	0.3831 (4)	0.0840 (17)	
H17A	0.7272	0.1399	0.3185	0.126*	
H17B	0.8906	0.1422	0.4042	0.126*	
H17C	0.8881	0.3101	0.3740	0.126*	

### Atomic displacement parameters (Å^2^)

**Table:**

	*U*^11^	*U*^22^	*U*^33^	*U*^12^	*U*^13^	*U*^23^
F1	0.0504 (14)	0.0478 (13)	0.0771 (17)	0.0064 (11)	0.0123 (12)	−0.0136 (12)
O1	0.077 (2)	0.081 (2)	0.0573 (19)	0.0394 (19)	0.0278 (17)	0.0012 (18)
O2	0.084 (2)	0.072 (2)	0.0611 (19)	0.0311 (18)	0.0186 (17)	−0.0087 (17)
O3	0.0514 (17)	0.0659 (18)	0.0658 (19)	0.0245 (15)	0.0267 (15)	0.0086 (15)
O4	0.0438 (15)	0.0543 (16)	0.0548 (17)	0.0115 (13)	0.0164 (13)	−0.0057 (13)
N1	0.0444 (19)	0.0450 (18)	0.056 (2)	0.0129 (15)	0.0172 (16)	0.0015 (16)
N2	0.0415 (18)	0.0460 (18)	0.0441 (18)	0.0137 (15)	0.0112 (14)	0.0023 (15)
N3	0.053 (2)	0.060 (2)	0.0391 (17)	0.0302 (17)	0.0041 (15)	−0.0071 (15)
N4	0.067 (2)	0.0451 (19)	0.052 (2)	0.0196 (17)	0.0221 (17)	−0.0016 (16)
C1	0.066 (3)	0.068 (3)	0.051 (3)	0.038 (2)	0.020 (2)	0.010 (2)
C2	0.059 (3)	0.048 (2)	0.036 (2)	0.029 (2)	0.0121 (18)	0.0061 (17)
C3	0.051 (2)	0.053 (2)	0.044 (2)	0.0278 (19)	0.0146 (19)	0.0114 (19)
C4	0.049 (2)	0.0373 (19)	0.040 (2)	0.0200 (17)	0.0092 (17)	0.0053 (16)
C5	0.042 (2)	0.045 (2)	0.055 (2)	0.0169 (18)	0.0115 (18)	0.0051 (19)
C6	0.042 (2)	0.038 (2)	0.056 (2)	0.0102 (17)	0.0063 (18)	−0.0031 (18)
C7	0.051 (2)	0.0363 (19)	0.046 (2)	0.0205 (17)	0.0110 (18)	0.0056 (17)
C8	0.042 (2)	0.046 (2)	0.044 (2)	0.0186 (17)	0.0121 (17)	0.0003 (18)
C9	0.042 (2)	0.057 (2)	0.059 (3)	0.0149 (19)	0.0090 (19)	−0.007 (2)
C10	0.054 (2)	0.048 (2)	0.038 (2)	0.0236 (19)	0.0104 (18)	−0.0031 (17)
C11	0.041 (2)	0.0352 (18)	0.0396 (19)	0.0131 (16)	0.0082 (16)	0.0025 (16)
C12	0.069 (3)	0.058 (3)	0.068 (3)	0.018 (2)	0.025 (2)	0.010 (2)
C13	0.066 (3)	0.068 (3)	0.052 (3)	0.031 (2)	0.001 (2)	−0.010 (2)
C14	0.083 (3)	0.070 (3)	0.049 (3)	0.032 (3)	0.010 (2)	−0.001 (2)
C15	0.063 (3)	0.079 (3)	0.057 (3)	0.034 (2)	0.015 (2)	−0.005 (2)
C16	0.063 (3)	0.061 (3)	0.053 (2)	0.038 (2)	0.015 (2)	0.005 (2)
C17	0.098 (4)	0.073 (3)	0.074 (3)	0.021 (3)	0.048 (3)	−0.007 (3)

### Geometric parameters (Å, º)

**Table:**

F1—C6	1.361 (4)	C5—C6	1.368 (5)
O1—C1	1.339 (5)	C5—H5	0.9300
O1—H1	0.83 (3)	C6—C7	1.407 (5)
O2—C1	1.210 (5)	C7—C8	1.395 (5)
O3—C3	1.267 (4)	C8—C11	1.401 (5)
O4—C8	1.377 (4)	C9—H9A	0.9700
O4—C9	1.448 (4)	C9—H9B	0.9700
N1—N2	1.434 (4)	C10—H10	0.9300
N1—C9	1.439 (5)	C12—H12A	0.9600
N1—C12	1.484 (6)	C12—H12B	0.9600
N2—C10	1.341 (4)	C12—H12C	0.9600
N2—C11	1.387 (4)	C13—C14	1.507 (6)
N3—C7	1.397 (4)	C13—H13A	0.9700
N3—C13	1.465 (5)	C13—H13B	0.9700
N3—C16	1.470 (5)	C14—H14A	0.9700
N4—C14	1.438 (6)	C14—H14B	0.9700
N4—C15	1.451 (5)	C15—C16	1.506 (5)
N4—C17	1.464 (5)	C15—H15A	0.9700
C1—C2	1.491 (5)	C15—H15B	0.9700
C2—C10	1.368 (5)	C16—H16A	0.9700
C2—C3	1.425 (5)	C16—H16B	0.9700
C3—C4	1.470 (5)	C17—H17A	0.9600
C4—C5	1.387 (5)	C17—H17B	0.9600
C4—C11	1.399 (5)	C17—H17C	0.9600
			
C1—O1—H1	106 (4)	H9A—C9—H9B	107.9
C8—O4—C9	111.3 (3)	N2—C10—C2	121.0 (3)
N2—N1—C9	106.7 (3)	N2—C10—H10	119.5
N2—N1—C12	109.9 (3)	C2—C10—H10	119.5
C9—N1—C12	113.8 (3)	N2—C11—C4	119.3 (3)
C10—N2—C11	122.3 (3)	N2—C11—C8	120.0 (3)
C10—N2—N1	118.4 (3)	C4—C11—C8	120.7 (3)
C11—N2—N1	119.1 (3)	N1—C12—H12A	109.5
C7—N3—C13	121.3 (3)	N1—C12—H12B	109.5
C7—N3—C16	117.4 (3)	H12A—C12—H12B	109.5
C13—N3—C16	110.8 (3)	N1—C12—H12C	109.5
C14—N4—C15	110.1 (3)	H12A—C12—H12C	109.5
C14—N4—C17	111.2 (4)	H12B—C12—H12C	109.5
C15—N4—C17	111.6 (4)	N3—C13—C14	108.0 (4)
O2—C1—O1	120.9 (4)	N3—C13—H13A	110.1
O2—C1—C2	123.9 (4)	C14—C13—H13A	110.1
O1—C1—C2	115.2 (4)	N3—C13—H13B	110.1
C10—C2—C3	121.6 (3)	C14—C13—H13B	110.1
C10—C2—C1	116.6 (3)	H13A—C13—H13B	108.4
C3—C2—C1	121.7 (4)	N4—C14—C13	111.6 (4)
O3—C3—C2	123.6 (3)	N4—C14—H14A	109.3
O3—C3—C4	120.3 (3)	C13—C14—H14A	109.3
C2—C3—C4	116.0 (3)	N4—C14—H14B	109.3
C5—C4—C11	118.8 (3)	C13—C14—H14B	109.3
C5—C4—C3	121.6 (4)	H14A—C14—H14B	108.0
C11—C4—C3	119.6 (3)	N4—C15—C16	111.0 (4)
C6—C5—C4	119.0 (4)	N4—C15—H15A	109.4
C6—C5—H5	120.5	C16—C15—H15A	109.4
C4—C5—H5	120.5	N4—C15—H15B	109.4
F1—C6—C5	118.2 (3)	C16—C15—H15B	109.4
F1—C6—C7	117.0 (3)	H15A—C15—H15B	108.0
C5—C6—C7	124.8 (3)	N3—C16—C15	109.4 (4)
N3—C7—C8	119.3 (3)	N3—C16—H16A	109.8
N3—C7—C6	125.6 (3)	C15—C16—H16A	109.8
C8—C7—C6	115.1 (3)	N3—C16—H16B	109.8
O4—C8—C7	118.6 (3)	C15—C16—H16B	109.8
O4—C8—C11	119.9 (3)	H16A—C16—H16B	108.2
C7—C8—C11	121.5 (3)	N4—C17—H17A	109.5
N1—C9—O4	112.4 (3)	N4—C17—H17B	109.5
N1—C9—H9A	109.1	H17A—C17—H17B	109.5
O4—C9—H9A	109.1	N4—C17—H17C	109.5
N1—C9—H9B	109.1	H17A—C17—H17C	109.5
O4—C9—H9B	109.1	H17B—C17—H17C	109.5
			
C9—N1—N2—C10	147.4 (4)	N3—C7—C8—C11	177.4 (4)
C12—N1—N2—C10	−88.8 (4)	C6—C7—C8—C11	−1.6 (6)
C9—N1—N2—C11	−36.1 (5)	N2—N1—C9—O4	62.1 (4)
C12—N1—N2—C11	87.7 (4)	C12—N1—C9—O4	−59.3 (4)
O2—C1—C2—C10	−3.8 (7)	C8—O4—C9—N1	−56.7 (4)
O1—C1—C2—C10	176.1 (4)	C11—N2—C10—C2	0.7 (6)
O2—C1—C2—C3	179.1 (4)	N1—N2—C10—C2	177.0 (4)
O1—C1—C2—C3	−1.0 (6)	C3—C2—C10—N2	−1.8 (6)
C10—C2—C3—O3	178.9 (4)	C1—C2—C10—N2	−178.9 (4)
C1—C2—C3—O3	−4.2 (6)	C10—N2—C11—C4	2.2 (6)
C10—C2—C3—C4	0.0 (6)	N1—N2—C11—C4	−174.1 (3)
C1—C2—C3—C4	177.0 (4)	C10—N2—C11—C8	−178.1 (4)
O3—C3—C4—C5	3.6 (6)	N1—N2—C11—C8	5.6 (5)
C2—C3—C4—C5	−177.5 (4)	C5—C4—C11—N2	176.4 (4)
O3—C3—C4—C11	−176.1 (4)	C3—C4—C11—N2	−3.9 (6)
C2—C3—C4—C11	2.8 (5)	C5—C4—C11—C8	−3.3 (6)
C11—C4—C5—C6	1.5 (6)	C3—C4—C11—C8	176.4 (4)
C3—C4—C5—C6	−178.2 (4)	O4—C8—C11—N2	2.0 (6)
C4—C5—C6—F1	178.3 (4)	C7—C8—C11—N2	−176.2 (4)
C4—C5—C6—C7	0.2 (7)	O4—C8—C11—C4	−178.3 (3)
C13—N3—C7—C8	−148.1 (4)	C7—C8—C11—C4	3.4 (6)
C16—N3—C7—C8	70.4 (5)	C7—N3—C13—C14	157.3 (4)
C13—N3—C7—C6	30.9 (6)	C16—N3—C13—C14	−58.9 (5)
C16—N3—C7—C6	−110.7 (5)	C15—N4—C14—C13	−59.0 (5)
F1—C6—C7—N3	2.7 (6)	C17—N4—C14—C13	176.7 (3)
C5—C6—C7—N3	−179.2 (4)	N3—C13—C14—N4	59.4 (5)
F1—C6—C7—C8	−178.3 (3)	C14—N4—C15—C16	57.3 (5)
C5—C6—C7—C8	−0.2 (6)	C17—N4—C15—C16	−178.7 (4)
C9—O4—C8—C7	−159.0 (4)	C7—N3—C16—C15	−156.2 (3)
C9—O4—C8—C11	22.7 (5)	C13—N3—C16—C15	58.4 (5)
N3—C7—C8—O4	−0.8 (6)	N4—C15—C16—N3	−56.9 (5)
C6—C7—C8—O4	−179.9 (4)		

### Hydrogen-bond geometry (Å, º)

**Table:**

*D*—H···*A*	*D*—H	H···*A*	*D*···*A*	*D*—H···*A*
O1—H1···O3	0.83 (3)	1.77 (2)	2.560 (4)	159 (5)
C12—H12*B*···F1^i^	0.96	2.62	3.422 (3)	140 (4)
C15—H15*B*···F1^ii^	0.97	2.54	3.446 (3)	155 (5)

Symmetry codes: (i) *x*+1, *y*+1, *z*; (ii) *x*+1, *y*, *z*.
